# Investigation of the Effect of Dimple Bionic Nonsmooth Surface on Tire Antihydroplaning

**DOI:** 10.1155/2015/694068

**Published:** 2015-08-20

**Authors:** Haichao Zhou, Guolin Wang, Yangmin Ding, Jian Yang, Huihui Zhai

**Affiliations:** ^1^Department of Transportation Engineering, School of Automotive and Traffic Engineering, Jiangsu University, Zhenjiang 212013, China; ^2^Civil and Environmental Engineering, School of Engineering, Rutgers, The State University of New Jersey, Piscataway, NJ 08854, USA

## Abstract

Inspired by the idea that bionic nonsmooth surfaces (BNSS) reduce fluid adhesion and resistance, the effect of dimple bionic nonsmooth structure arranged in tire circumferential grooves surface on antihydroplaning performance was investigated by using Computational Fluid Dynamics (CFD). The physical model of the object (model of dimple bionic nonsmooth surface distribution, hydroplaning model) and SST *k* − ω turbulence model are established for numerical analysis of tire hydroplaning. By virtue of the orthogonal table L_16_(4^5^), the parameters of dimple bionic nonsmooth structure design compared to the smooth structure were analyzed, and the priority level of the experimental factors as well as the best combination within the scope of the experiment was obtained. The simulation results show that dimple bionic nonsmooth structure can reduce water flow resistance by disturbing the eddy movement in boundary layers. Then, optimal type of dimple bionic nonsmooth structure is arranged on the bottom of tire circumferential grooves for hydroplaning performance analysis. The results show that the dimple bionic nonsmooth structure effectively decreases the tread hydrodynamic pressure when driving on water film and increases the tire hydroplaning velocity, thus improving tire antihydroplaning performance.

## 1. Introduction

Safe operation on wet road is one of the major concerns of pavement engineers and researchers. It is reported that approximately 20% of all road traffic accidents occur in wet weather conditions and most of the traffic accidents are related to tire performance [1]. Tire hydroplaning is one of the top five incentives for traffic accidents. When vehicle maneuvers at a certain speed on a wet road, once the vertical effort generated by the hydrodynamic pressure resulting from the contact area exceeds the tire weight, the contact between tire and road is destroyed by a fluid film and hydroplaning is formed [2, 3]. Driving under such conditions is hazardous. As the only part by which the vehicle interacts with the road surface, the tire tread determines the comprehensive performance of tire, such as noise, wear, and hydroplaning. So it is critically important to improve tire antihydroplaning performance. Experimental work and theoretical analysis have brought in some innovations, such as pavement grooving, which permits at least partial elimination of hydroplaning. Based on experimental investigations and imaging technology, researchers used glass plate to present visual images of tire contact shape in water [4]. Unfortunately, this kind of experimental method requires tire manufacture and test setup, which is more time consuming and costly. For analytical theory, there are still some complicated problems in analysis of hydroplaning phenomenon. For example, the fluid flow system is nonlinear and there is no accurate mathematical model for tire surface deformation. Thus, it is impossible to formulate a description of tire hydroplaning.

The rapid development of computer and numerical simulation technology provides necessary technical support for analyzing tire antihydroplaning. Aksenov et al. [5] presented a three-dimensional simulation of the interaction between tire and free surface flow by virtue of Computational Fluid Dynamics (CFD) technology, but the deformation of tire surface was ignored and the computational domain remained fixed in time in his work. Grogger and Weiss [6] pointed out that deformed tire had significant influence on hydrodynamic pressure at higher vehicular speeds. Simulated several patterned tires and compared tire contact forces by Cho et al. [7] showed that the tread patterns structure parameter determines anti-hydroplaning performance. Fwa et al. [8, 9] studied the effects of vertical and horizontal circumferential groove dimension on hydroplaning, indicating that unit tread pattern area can be used as evaluation of the performance of hydroplaning. Normally, increasing void in tread pattern circumferential grooves can provide additional space to absorb rainwater and increase force to cut water film. This method indeed increases the tire antihydroplaning performance, but it comes at a cost of other performance factors, as improving one may degrade the other performance factors. Wies et al. [10] discovered that 1% improvement of hydroplaning by increasing circumferential grooves void will lead to 0.6% reduction in vehicle stability, 0.4% increase in rolling resistance, 2.3% deduction in rolling noise, and so on. Obviously, the traditional method of improving tire antihydroplaning performance will result in reduction of other tire performances. Therefore, it is significant to find other methods for improving tire anti-hydroplaning with pattern circumferential grooves volume unaltered.

From researching the surface characteristics of nature animals, biologists find that bionic nonsmooth surfaces (BNSS) can reduce fluid adhesion and resistance. NASA Research Center spearheaded the study of the surface structure of shark skin in the 1970s and found that the sharks' skin surface has microcircumferential groove structure distributed all over the body that can reduce the resistance of the shark's high-speed underwater swimming. Then, bionic principles were presented and applied in engineering domain [11, 12]. Great achievements for bionic applications have come out; Tian et al. [13] experimentally investigated the centrifugal pump with a concave dimple surface called a bionic coupling centrifugal pump (BCCP), and the results show that the efficiency of BCCPs obviously improved and the efficiency curve became more compressed than that of a conventional centrifugal pump over the effective working range. Song et al. [14] designed a dimple bionic nonsmooth surface on motor vehicle body panels; the results show that comparison with smooth surface and properly designed nonsmooth surfaces can have greater effects on drag reduction. Inspired by the bionic design, a new method for improving tire antihydroplaning is proposed in this paper, which applies the dimple bionic nonsmooth surface to reduce tire pattern circumferential grooves water flow resistance in the footprint and increase flow rate to improve hydroplaning velocity.

First, the hydroplaning velocity of the circumferential grooves tire was simulated with the FLUENT software. Then, tire hydroplaning velocity was compared with NASA hydroplaning equation and experimental data from Okano and Koishi [15] in order to illustrate hydroplaning model computing efficiency and validity. Secondly, with the help of an orthogonal table L_16_(4^5^), the parameters of dimple bionic structure design compared to the smooth structure were analyzed, and the priority level of the experimental factors as well as the best combination within the scope of the experiment was obtained. Finally, arranging the optimized dimple bionic nonsmooth structure on the bottom of the circumferential grooves, CFD technology is used to predict tire antihydroplaning performance. The present research shows that the tire pattern circumferential grooves bionic design of nonsmooth structures is one of the most feasible ways to improve antihydroplaning.

## 2. Computational Details

The traditional simulations of tire hydroplaning focus on the Coupled Eulerian-Lagrangian (CEL) method which can acquire tire deformation and lift force correctly. However, the water movement cannot be investigated in detail due to the arithmetic deficiency. Meanwhile, the calculation is time consuming. However, the CFD technique can smoothly solve the coupling problem and successfully deal with flow problems, and the free interface between air and water can be tracked by the Volume of Fluid (VOF) model.

### 2.1. Tire Model

**Figure 1:**
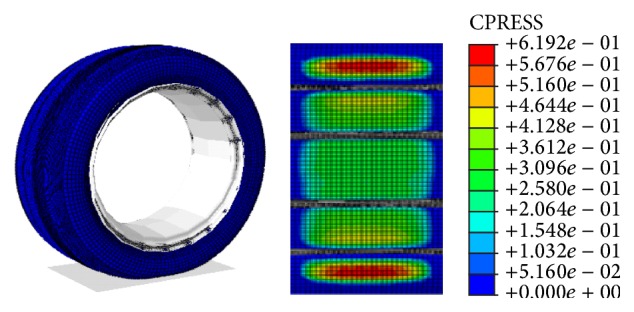
Tire footprint and contact deformation of tire under standard loading.

The structure of tire circumferential grooves determines tire drainage capability. The deformation of tire tread under load affects the drainage space. To analyze the water flow field in circumferential grooves in the tire-ground contact, the shape of the deformed tire and the contact pressures under normal load and pressure must be known. The finite element model (FEM) of a tire (205/50R16) was established, and the tire deformation was acquired with the wheel load of 4000 N and the inflation pressure of 200 kPa. The tire was not supposed to rotate; thus no centrifugal load was applied. Under this assumption, the tire static contact pressure is simulated. The comparison of simulation and experimental results is presented in Figure 1, and the method of tire print experiment is provided in [16]. The design of the circumferential grooves is in accordance with the testing tire, in which the breadth and the depth are 8 mm and 9 mm, respectively.

### 2.2. Hydroplaning Computational Domain Definition

**Figure 2:**
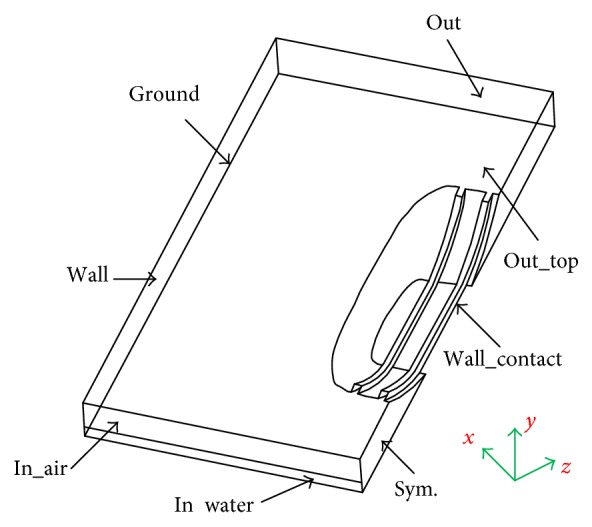
The computational model and boundary conditions.

When hydroplaning occurs, both water and air flow from the tire-pavement contact area. First, a fluid model which contains both water film and air flow is established, and then the geometric outer contour of the tire after rolling deformation is created, and the rolling tire model is removed during the fluid calculation. Simulation results may vary by about 5%–10% with the computation domain size. The domain size with height 50 mm, length in front of tire 300 mm and behind 600 mm, and width 350 mm is considered from previous studies [17].  Figure 2 shows the computational domain, which is meshed by using multiblock grid technique. The computational domain is discredited into five-sided structure mesh hydrides with unstructured tetrahedral mesh. The mesh in the front of the contact area and within the tread circumferential groove was refined, to ensure there are at least eight discrete units in a single tread circumferential groove. The whole grid is made up of 1,358,587 cells and 1,272,427 nodes. The simulation uses half tire model, and the water and air flow direction is in *Z*-direction.

### 2.3. VOF Model

**Figure 3:**
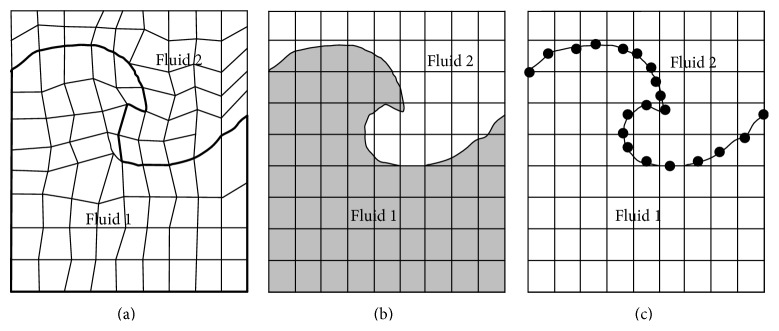
Three approaches to compute free surface flows: (a) surface fitting, (b) surface capturing, and (c) surface tracking.

In multiphase flow, the identifiable class of material is a phase that has a particular inertial response to and interaction with the flow and the potential held in which it is immersed. The proposed model is essentially a free surface flow with moving boundary. Generally, there are three approaches to solve this difficulty and compute free surface flows, namely, (i) surface fitting method, (ii) surface capturing method, and (iii) surface tracking method. Schematic representations of the three approaches are given in Figure 3. The major advantages and drawbacks of these three methods are discussed by Zhao et al. [18].

VOF method, which keeps and updates the field of volume fraction of one fluid in each cell instead of surface height, could be utilized to solve the advection equation of the volume fraction and predict the fluid interface accurately. In VOF model, fluids are not considered to be penetrating. For each additional phase in the model, a variable (volume fraction of the phase) is introduced in the computational cell. In every control volume, the volume of all phases must amount to unity. Alternatively, variables and properties of a given cell are purely representative of one phase or a mixture phase, depending on the volume fraction values.

The volume fraction equation in the cell is denoted by *α*_*q*_; where

$\;\alpha_{q}=0$ : the cell is empty (of the *q*th fluid);
 
*α*_*q*_ = 1: the cell is full (of the *q*th phase);
 0 < *α*_*q*_ < 1: the cell contains an interface between the *q*th fluid and one or more other fluids; based on the local value of *α*_*q*_, the appropriate properties and variables will be assigned to each control volume within the domain.

The continuity equation, for that particular fluid's volume fraction, is then solved, followed by the momentum equation. The primary phase volume fraction can be computed based on the constraint shown in (1)–(3). After the momentum equation is solved throughout the domain, the resulting velocity field and other quantities are shared among the phases, and thus tracking of the interface (volume fraction of each fluid) between phases is done. Consider

(1) ∂αq∂t+v∇αq=0,

(2) ∑q=1nαq=1,

(3) ∂∂tρv+∇·ρvv=−∇p+∇·μ∇v+∇vT+ρg+F,

where *v* is velocity; *g* is gravitation force vector; *t* is time; and *F* is the force vector due to external source. For additional scalars, such as turbulence quantities, a single set of transport equations is solved and the quantities are shared by the phases throughout the field.

The density *ρ* and the molecular viscosity *μ* in the equations are dependent on the volume fractions of all phases:

(4) μ=∑αqμq,μ=∑αqμq.

### 2.4. Control Equation and Turbulence Model

At present, for turbulence simulation analysis, there are mainly three turbulence control equations: the direct numerical simulation (DNS), the large eddy simulation (LES), and the Reynolds-averaged numerical simulation (RANS). In the three turbulent numerical simulation algorithms, Reynolds-averaged numerical simulation method perfectly reflects the swirl distribution within the boundary layer and other microflow information, including the merit of low computational demand and high efficiency; thus the Reynolds-averaged numerical simulation (RANS) is adopted in this paper. Equation (5) is the continuity equation and (6) is the Reynolds-averaged Navier-Stokes equation. Hence,

(5) ∂ρui∂xi=0,

(6) ∂ρuiuj∂xj=−∂p∂xi+∂∂xjμ∂ui∂uj+∂uj∂ui−23δij∂ui∂xi+∂τij∂xj,

where *ρ*, *μ* are the fluid density and the coefficient of the molecular viscosity, respectively. *u*_*i*_ is the mean velocity components. *x*_*i*_ is the Cartesian coordinates. *P* is the static pressure. *δ*_*ij*_ is the Kronecker delta.

SST  *k*-*ω* model [19] is chosen for the reason of consolidating the advantages of high Reynolds number model and the low Reynolds number model, utilizing mixed function to achieve gradual transition from standard model within the boundary layer to high Reynolds number model outside the boundary layer, and making the transition from the near-wall region to the full development region more perfect along with the higher calculation accuracy. Equation (7) is the turbulent kinetic energy equation, and (8) is the turbulence dissipation rate equation. They are given as follows:

(7) ∂ρκ∂t+∂ρκui∂xi=∂∂xjΓk∂κ∂xj+G~κ−Yκ+Sκ,

(8) ∂ρω∂t+∂ρκui∂xi=∂∂xjΓw∂ω∂xj+Gω−Yω+Dω+Sω,

where ${\tilde{G}}_{\kappa }$ is the generation of turbulence kinetic energy due to the mean velocity gradients; *G*_*ω*_ is the generation of *w*; Γ_*κ*_ and Γ_*ω*_ are the effective diffusivities of *k* and *ω*, respectively; *Y*_*κ*_ and *Y*_*ω*_ are the dissipation values of *k* and *ω*; *D*_*ω*_ is the cross-diffusion term; and *S*_*κ*_ and *S*_*ω*_ are source terms.

Pressure implicit with splitting of operators (PISO) is based on higher degree of approximate relation between the corrections for pressure and velocity. This study combines PISO algorithm, which can greatly decrease iteration numbers required for convergence, with an implicit and second-order accurate scheme to get the time-advanced solution.

### 2.5. Boundary Conditions

The hydroplaning phenomenon on a locked tire sliding on a flooded smooth pavement is modeled in this paper. For an observer in stationary frame of reference, the hydroplaning phenomena can be viewed as the tire at speed *U* m/s sliding along a smooth pavement flooded with water. Alternatively, in a moving wheel frame of reference, the hydroplaning phenomena can be viewed as a jet with a layer of air and a layer of water, entering on tire surface and pavement surface also moving at a speed of *U* m/s towards the tire surface. The hydroplaning model has been modeled in a moving frame of reference in this paper.

Figure 2 demonstrates the boundary conditions of the computational domain. As for a two-phase flow, the inlet has two similar types of boundary conditions for water and air. The lower part of 10 mm height stands for water and the rest stands for air, and inlet velocity of both water and air is 80 km/h. The outlet condition is defined as one atmospheric pressure. The friction produced by the fixed wall and the deflection of tread pattern circumferential grooves generated by flowing water force is neglected. The longitudinal plane in the center of the tire forms the symmetry plane.

## 3. Hydroplaning Results and Discussions

**Figure 4:**
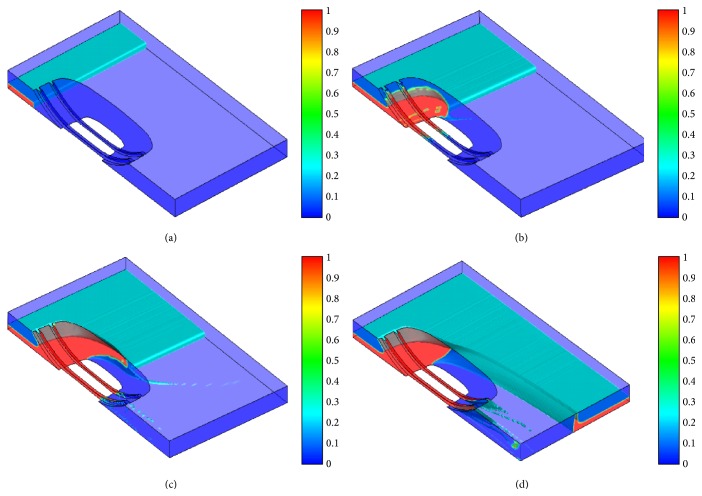
Free surface of water in tire hydroplaning at different times: (a) *t* = 0.005 s; (b) *t* = 0.01 s; (c) *t* = 0.015 s; (d) *t* = 0.04 s.

Figure 4 shows the interfacial velocities and void fractions on the ground, where the deep grey part represents water flow. It can be seen that flow separates and wave forms at the front of tire and water drains from the tire tread circumferential grooves, which is consistent with the reality.

**Figure 5:**
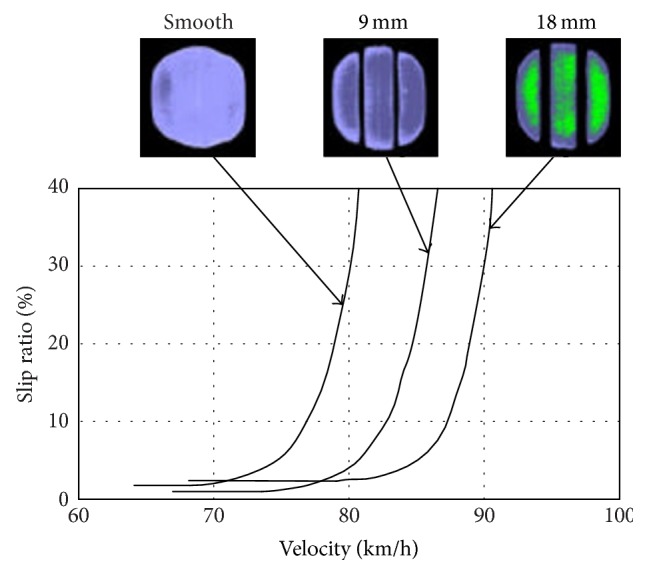
Relationships among slip ratio, velocity, and tread pattern obtained by experiment.

Okano and Koishi [15] conducted an experiment about tire hydroplaning, as shown in Figure 5. In the experiment, tires with different tread patterns were assembled to the same car, and then the car ran on a proving ground at certain acceleration. High-speed photography was used to shoot the whole process. One of their tires (the middle tire) is the same as the tire used in this paper. From the experiment results, it is noted that tire hydroplaning occurs when vehicle is moving at about 82 km/h. Comparison between experiment and simulation results shows that hydroplaning critical velocity obtained from this work is in reasonable agreement with experiment results.

## 4. Investigation on Tire Hydroplaning of Dimple Bionic Nonsmooth Surface

The physical model, governing equation, computational domain, and boundary conditions are the same as the hydroplaning model mentioned above except that dimple bionic nonsmooth surfaces are arranged on tire pattern circumferential grooves surfaces.

### 4.1. Dimple Bionic Nonsmooth Structure Model

According to the fluid boundary layer theory, the formula for estimating the boundary layer thickness is

(9) $$\begin{array}{c} \delta =0.37x{Re}_{x}^{-1/5}, \end{array}$$

where *x* is the flow displacement and Re_*x*_ is Reynolds number at corresponding *x* displacement. According to the structure parameters of tread pattern circumferential groove, it can be estimated that the boundary layer thickness is 0.9 mm. To make sure that the nonsmooth structure affects the internal structure of the boundary layer, its size should not be too close to the thickness of the boundary layer [17], so the depth of the dimple must be within this range.

**Figure 6:**
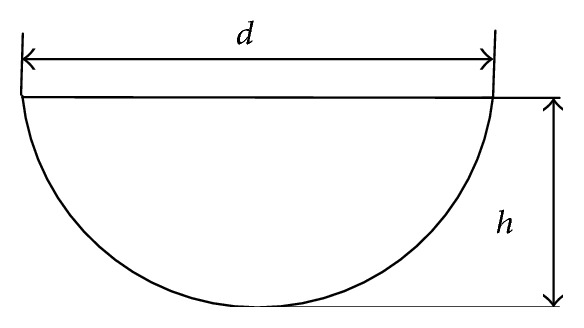
Sectional view of the dimple bionic nonsmooth symmetry plane.

**Figure 7:**
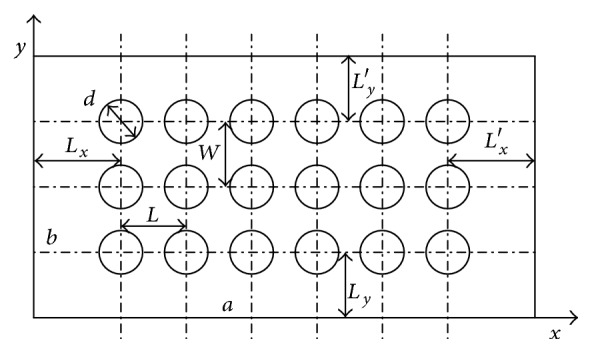
Distribution of dimple bionic nonsmooth surface.

**Figure 8:**
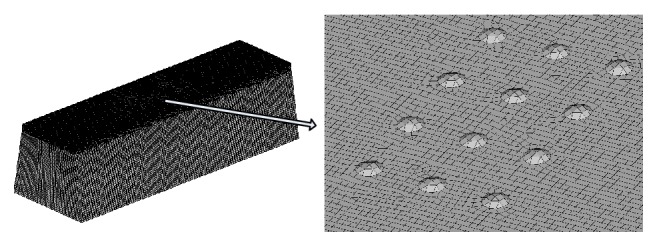
The mesh and enlarged view profile.

It is assumed that the dimple bionic nonsmooth surface is a sphere that intersects with the plane and the depth is *h*.  Figure 6 shows a sectional profile, where *d* is the diameter of the circle plane which intersects with the top of the dimple surface formed. The rectangle-profile dimple structure is arranged under the bottom of the pattern ditch as shown in Figure 7. To avoid the entrance effects and analyze the impact on the downstream flow field, the dimple structure is arranged in the central region of the circumferential groove shown in Figure 8.

*L* is distance between centers along the *x* direction, *W* is distance between centers along the *y* direction, length direction of the model, *a* is the length of the model in *x* direction, and *b* is the width of the model in *y* direction. The definition for *L*_*x*_, *L*_*x*_′, *L*_*y*_, and *L*_*y*_′ is shown in Figure 7. It is assumed that the dimple bionic nonsmooth structure has a symmetric distribution along the *y* direction; thus *L*_*y*_ = *L*_*y*_′.

### 4.2. Model Meshing and Solver Settings

According to the structure parameters of circumferential groove, the simulation model is 30 mm × 7.5 mm in the *x* direction and *y* direction, respectively. Once selected the size of simulation model and dimple, using combine tetrahedral and hexahedral grid discrete the model to meet the mesh requires, and local grid refinement nearby the wall.

Viscous boundary layer is 0 ≤ *y* ^+^ ≤ 5, thus the first mesh close to the wall must be within *y* ^+^ ≤ 5, and the thickness of the first layer mesh can be obtained by *y* = *μy* ^+^/*C*_*μ*_^1/4^ *k*_*p*_^1/2^, where *y* ^+^ is dimensionless distance to the wall, *μ* is the dynamic viscosity of the fluid, *C*_*μ*_ is the empirical constant (0.09), and *k*_*p*_ is the turbulent kinetic energy for the first node *P*. In addition, for the SST  *κ*-*ω* model, the minimum number of meshes in the boundary layer is 15. After repeated attempts, the simulation uses a mesh size of 0.01 mm, an increase of 1.2; maximum mesh size in the computational domain is 0.2 mm. Figure 8 shows the mesh and an enlarged view of the dimple bionic nonsmooth surface mesh, respectively.

### 4.3. Analysis of Orthogonal Experiment

In order to explore the effect of dimple bionic nonsmooth structure and distribution forms on drag reduction, the orthogonal design method was used for simulation arrangement. The drag reduction rate *R* is expressed as follows:

(10) $$\begin{array}{c} R=\frac{C_{y}-C_{n}}{C_{y}}\times 100\%, \end{array}$$

where *C*_*y*_ = *F*_*y*_/(1/2*ρv* ^2^ *S*) and *C*_*n*_ = *F*_*n*_/(1/2*ρv* ^2^ *S*) are the drag coefficient of the smooth model and the dimple bionic nonsmooth surface model, respectively, *F*_*y*_ and *F*_*n*_ are the flow resistance of the original model and the dimple bionic nonsmooth surface model, *ρ* is flow density, *v* is velocity, and *S* is the projected area which is perpendicular to the flow direction. *R* ≥ 0 indicates that the dimple bionic nonsmooth surface model has drag reduction effects; otherwise, *R* ≤ 0, indicating that the model does not have a drag reduction, and the larger the absolute value, the more obvious the drag reduction or drag increase.

L_16_(4^5^) orthogonal arrangement experiment is selected.  Table 1 shows the factors for orthogonal experiment, where *A*, *B*, *C*, *D*, and *E*, respectively, indicate circle diameter at the top of the dimple (*d*/mm), dimple depth (*h*/mm), dimple downstream spacing (*L*/mm), dimple lateral spacing (*W*/mm), and the flow velocity (*v*/(km/h)). The level of each factor is four. The orthogonal test results are shown in Table 2.

According to the test results in Table 2, the results of the range analysis are shown in Table 3, where $\bar{T_{1j}}$, $\bar{T_{2j}}$, $\bar{T_{3j}}$, and $\bar{T_{4j}}$ are, respectively, the average results corresponding to *j*th column and *R*_*j*_ is the range, by which the effects of different factors on drag effects can be estimated. As shown in Table 3, *R*_*B*_ > *R*_*A*_ > *R*_*E*_ > *R*_*C*_ > *R*_*D*_; namely, the effect order of the five factors on the dimple bionic nonsmoothed surface of drag reduction is *B* > *A* > *E* > *C* > *D*. Furthermore, the level of each factor can be determined by comparing $\bar{T_{ij}}\;\;(1\leq i\leq 4)$. It can be seen from Table 3 that *B*_4_, *A*_1_, *E*_1_, *C*_4_, and *D*_3_ are preferred level of each factor. When the top circle diameter of the dimple is 0.3 mm, with the depth of 1/8*d*, the flows spacing is 3*d*, the lateral spacing is 2 mm, and the drag reduction at each flow velocity is optimum dimple surface.

### 4.4. Analysis of Resistance Reduction

#### 4.4.1. Wall Shear Stress Analysis

**Figure 9:**
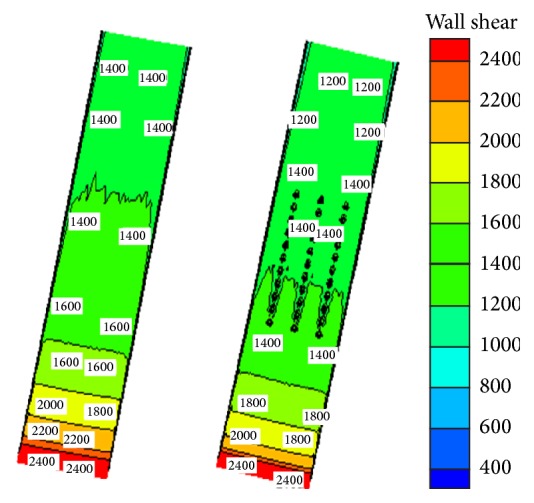
Comparison of wall shear stress.

As a result of the interaction between the fluid and the wall, wall shear stress reflects the value of the viscous resistance.  Figure 9 is the comparison of shear stress between the original and optimized dimple nonsmooth surface model with water flow velocity at 90 km/h. It can be seen from the figure that the stress in the front entrance is almost the same for the two models. In the center of the model, due to the presence of nonsmooth structure of the dimple surface, the wall shear stress is significantly smaller than the smooth surface. At the end of the model, the wall shear stress on the dimple bionic nonsmooth surface is still smaller than that on a smooth surface. Thus, the dimple bionic nonsmooth surface not only reduces the wall shear stress, but also reduces the wall shear stress downstream of the dimple bionic nonsmooth surface.

**Figure 10:**
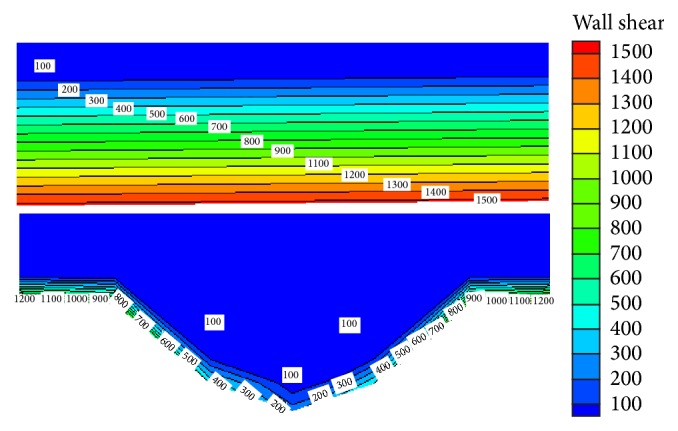
Comparison of shear stress at *x* = 15 mm.

Figure 10 shows the shear stress of the dimple bionic nonsmooth model *x* = 15 mm cross section. As shown in Figure 10, the shear stress in the bottom of the smooth surface maintains 1500 Pa; the farther the distance from the wall is, the smaller the shear stress is, which eventually stabilizes at 100 Pa. However, for dimple bionic nonsmooth surface, it can be seen that near the dimple bionic nonsmooth wall the shear stress is significantly less than that in the smooth surface, especially in the dimple bionic nonsmooth area, where the shear stress is significantly reduced, and the closer the bottom of the dimple bionic nonsmooth surface is, the smaller the wall shear stress is. Therefore, due to the presence of dimple bionic nonsmooth structure, the wall shear stress is significantly reduced, and resistance when the water flows through the tire tread circumferential grooves is smaller; thus the water can flow more smoothly through the circumferential groove.

#### 4.4.2. Velocity Field Analysis

**Figure 11:**
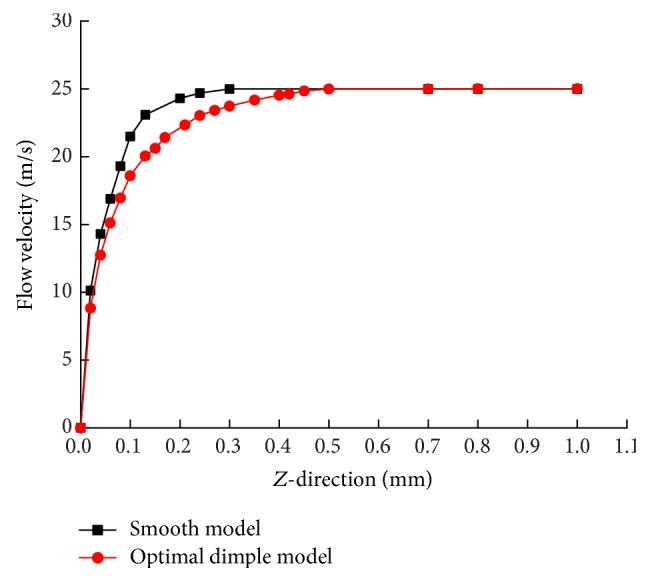
Comparison of velocity gradient.

Figure 11 shows the comparison chart of velocity of a characteristic plane at cross section *x* = 15, *z* = 0 ~ 1 mm height between the smooth surface and dimple surface. As shown in Figure 11, the smooth model at 0.3 mm height reached mainstream velocity, and the optimal dimple model reached the mainstream speeds at 0.5 mm height, so the velocity gradient changes in the smooth model are larger than in the dimple bionic nonsmooth model. According to (11), the viscous resistance is proportional to the relationship between the velocity gradients, so the dimple bionic nonsmooth surface viscous drag is smaller than a smooth surface. In other words, dimple is equivalent to increasing the thickness of the boundary layer. As a result, the frictional resistance is reduced and the water can flow through the tire grooves more easily. Consider

(11) $$\begin{array}{c} \tau =\tau_{w}+\tau_{t}=\mu \frac{\partial v_{x}}{\partial y}+\mu_{t}\frac{\partial {\bar{v}}_{x}}{\partial y}. \end{array}$$

#### 4.4.3. Velocity Vector Analysis

**Figure 12:**
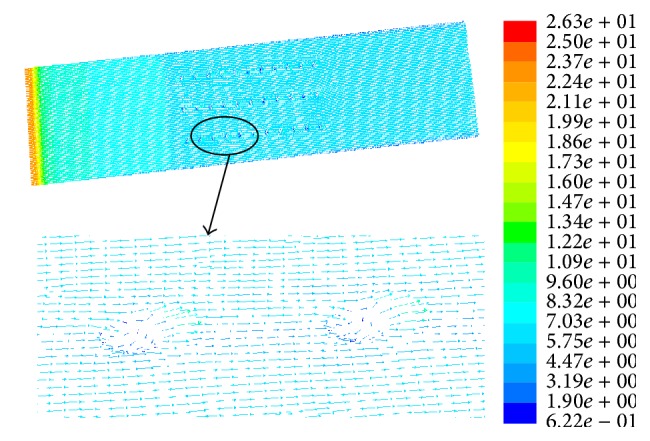
Velocity vector of dimple bionic nonsmooth surface.

**Figure 13:**
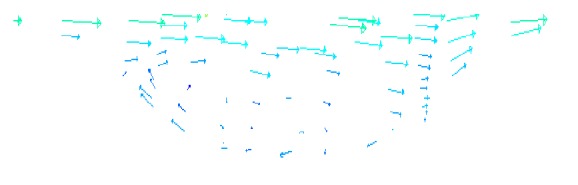
Velocity vector within dimple bionic nonsmooth surface.

Figure 12 is the surface velocity vector diagram of the optimal dimple bionic nonsmooth model under 90 km/h.  Figure 13 shows the velocity vector inside the dimple. As can be seen from Figure 13, there is a low-speed rotation inside the dimple, and the water flows inside the surface rather than over the top; therefore the water frictional resistance is decreased. Flow direction at the bottom of the dimple is opposite to the upper flow direction, while flow direction on the top is in the same water direction, which forms “Rolling Bearing” effects, making sliding contact with the wall of the fluid flow into rolling contact with the fluid, thus reducing the frictional resistance. Besides, the friction direction of the low-speed flow at the bottom is in line with the flow direction, which produces an additional push fluid effect and ultimately reduces the viscous friction.

## 5. Tire Hydroplaning Analysis

**Figure 14:**
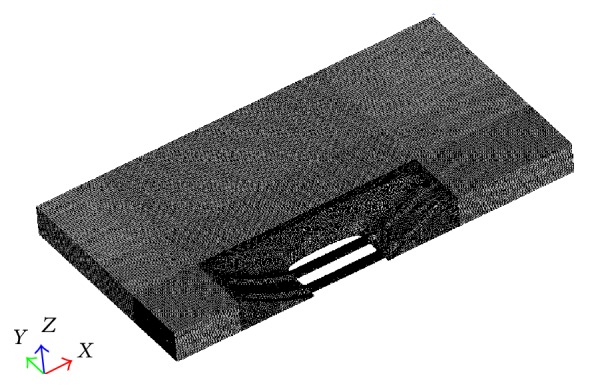
Hydroplaning computational domain mesh.

The optimized dimple structure through orthogonal experiment is arranged at the bottom of the circumferential groove, and comparison is made with the original smooth structure after the CFD analysis of tire hydroplaning. Considering the symmetry of the model, half of the tire footprint region is used for analysis.  Figure 14 shows the half tire hydroplaning model arranged with dimple bionic nonsmooth structure. A meshing method of combining structured grid and unstructured grid is adopted, and the turbulence model, boundary conditions, and other settings are the same as those of single grooves analysis.

Tire groove drainage capacity has a significant impact on tire performance. In general, the stronger the drainage circumferential grooves, the better the antihydroplaning performance of the tire.  Table 4 shows the mass flow per unit area of the original model and the dimple bionic nonsmooth model. As can be seen from the table, the dimple bionic nonsmooth structure reduces flow resistance; the outlet flow compared to the original model at each flow velocity has been improved.  Table 5 presents the hydrodynamic pressure acting on tire tread for the original model and the dimple bionic nonsmooth model, at 70 km/h, 80 km/h, 90 km/h, and 100 km/h.

The hydrodynamic pressure can be used as the evaluation of tire hydroplaning performance. HORNE proposed that when hydrodynamic pressure acting on the tire tread is greater than the tire inflation pressure, tire hydroplaning occurs [2]. Combined with the predicted values in Table 5, with curve fitting, the relationship between the hydrodynamic pressure and speed for the original model (*y*_1_) and the dimple bionic nonsmooth model (*y*_2_) is obtained as follows:

(12) y1=0.0314v2−0.1368v+0.4554,y2=0.0305v2−0.1417v+0.2961.

And can be seen from *y*_1_ and *y*_2_, the hydrodynamic pressure is proportional to the square of tire speed, and therefore the relationship between hydrodynamic pressure of the CFD model and the flow speed meets the basic theory *P* = 1/2*ρv* ^2^, and thus the model established in this paper is validated. Given inflation pressure 240 kPa, the critical hydroplaning speed is 89.58 km/h and 90.8 km/h, respectively, for the original model and dimple bionic nonsmooth surface model. Obviously, the dimple bionic nonsmooth surface arranged at the bottom of groove improves the tire antihydroplaning performance by increasing the critical hydroplaning speed by 1.22 km/h.

## 6. Conclusions

1. There are “Rolling Bearing” effects inside the dimple surface. The water flows inside the surface rather than over the top; therefore the water frictional resistance decreases, and the groove water draining capacity is improved.
2. The arrangement of dimple bionic nonsmooth surface on the bottom of tire pattern circumferential grooves has an impact on reducing the water resistance flowing through the circumferential grooves as well as improving the circumferential grooves drainage capacity. The depth of dimple, the top circle diameter of dimple, and the spacing between the adjacent dimples all have influence on the drag reduction effect. However, the depth of dimple has the greatest impact on the drag reduction.
3. Dimple bionic nonsmooth surface can reduce the wall shear stress and decrease the velocity gradient perpendicular to the flow direction along with the frictional resistance of the surface. The tread dynamic pressure can be decreased when driving on the water surface by the method of arranging the dimple bionic nonsmooth structure on the bottom of tire pattern circumferential grooves. Therefore, it can improve the tire antihydroplaning performance.

## Figures and Tables

**Table 1:** Orthogonal test factors.

Level	Factors				
	*A*	*B*	*C*	*D*	*E*
1	0.3	1/2*d*	1.5*d*	1	70
2	0.4	1/4*d*	2*d*	1.5	80
3	0.5	1/6*d*	2.5*d*	2	90
4	0.6	1/8*d*	3*d*	2.5	100

**Table 2:** Orthogonal test results.

Test number	Factors					*F*_*n*_ × 10^−3^/N	*F*_*y*_ × 10^−3^/N	*R*/%
	*A*	*B*	*C*	*D*	*E*			
1	1	1	1	1	1	231.35	231.96	0.26
2	1	2	2	2	2	289.05	291.45	0.82
3	1	3	3	3	3	348.69	358.76	2.81
4	1	4	4	4	4	416.86	429.83	3.02
5	2	1	2	3	4	439.43	429.83	−2.23
6	2	2	1	4	3	374.62	358.76	−4.42
7	2	3	4	1	2	282.72	291.45	3.00
8	2	4	3	2	1	221.51	231.96	4.51
9	3	1	3	4	2	300.38	291.45	−3.06
10	3	2	4	3	1	229.59	231.96	1.02
11	3	3	1	2	4	439.56	429.83	−2.26
12	3	4	2	1	3	352.93	358.76	1.63
13	4	1	4	2	3	376.23	358.76	−4.87
14	4	2	3	1	4	452.79	429.83	−5.34
15	4	3	2	4	1	228.67	231.96	1.42
16	4	4	1	3	2	288.06	291.45	1.16

**Table 3:** Range analysis of experiment results.

Test	Factors				
	*A*	*B*	*C*	*D*	*E*
$\bar{T_{1j}}$	1.73	−2.48	−1.32	−0.11	1.80
$\bar{T_{2j}}$	0.22	−1.98	0.41	−0.45	0.48
$\bar{T_{3j}}$	−0.67	1.24	−0.27	0.69	−1.21
$\bar{T_{4j}}$	−1.91	2.58	0.54	−0.76	−1.70
*R*_*j*_	3.64	5.06	1.86	1.45	3.50

**Table 4:** Comparison of outlet flow at different speeds.

	70 km/h	80 km/h	90 km/h	100 km/h
Smooth surface model (kg/m^2^·s)	19365.4	22160.3	24893.6	27749.9
Dimple surface model (kg/m^2^·s)	19598.7	22428.4	25207.3	28077.5
Flow change/%	1.04	1.13	1.16	1.09

**Table 5:** Comparison of hydrodynamic pressure at different speeds.

	70 km/h	80 km/h	90 km/h	100 km/h
Smooth surface model (kPa)	145.33	190.2	241.68	302.29
Dimple surface model (kPa)	140.86	184.38	233.74	292.46

## References

[1] Murad M. M., Abaza K. A. (2006). Pavement friction in a program to reduce wet weather traffic accidents at the network level. *Transportation Research Record*.

[2] Horne W. B., Dreher R. C. (1963). *Phenomena of Pneumatic Tire Hydroplaning*.

[3] Vincent S., Sarthou A., Caltagirone J.-P. (2011). Augmented Lagrangian and penalty methods for the simulation of two-phase flows interacting with moving solids. Application to hydroplaning flows interacting with real tire tread patterns. *Journal of Computational Physics*.

[4] Suzuki T., Fujikawa T. (2001). Improvement of hydroplaning performance based on water flow around tires. *SAE Transactions*.

[5] Aksenov A., Dyadkin A., Gudzovsky A. Numerical simulation of car tire aquaplaning.

[6] Grogger H., Weiss M. (1997). Calculation of the hydroplaning of a deformable smooth-shaped and longitudinally-grooved tire. *Tire Science and Technology*.

[7] Cho J. R., Lee H. W., Sohn J. S., Kim G. J., Woo J. S. (2006). Numerical investigation of hydroplaning characteristics of three-dimensional patterned tire. *European Journal of Mechanics—A/Solids*.

[8] Fwa T. F., Pasindu H. R., Ong G. P. (2012). Critical rut depth for pavement maintenance based on vehicle skidding and hydroplaning consideration. *Journal of Transportation Engineering*.

[9] Kumar S. S., Anupam K., Fwa T. F. (2010). Analyzing effect of tire groove patterns on hydroplaning speed. *Journal of the Eastern Asia Society for Transportation Studies*.

[10] Wies B., Roeger B., Mundl R. (2009). Influence of pattern void on hydroplaning and related target conflicts. *Tire Science and Technology*.

[11] Tian L.-M., Ren L.-Q., Liu Q.-P., Han Z.-W., Jiang X. (2007). The mechanism of drag reduction around bodies of revolution using bionic non-smooth surfaces. *Journal of Bionic Engineering*.

[12] Wang G.-L., Zhou H.-C., Yang J., Liang C., Jin L. (2013). Influence of bionic non-smooth surface on water flow in antiskid tire tread pattern. *Journal of Donghua University (English Edition)*.

[13] Tian L., Gao Z., Ren L., Han Z., Liao G. (2013). The study of the efficiency enhancement of bionic coupling centrifugal pumps. *Journal of the Brazilian Society of Mechanical Sciences and Engineering*.

[14] Song X.-W., Zhang G.-G., Wang Y., Hu S.-G. (2011). Use of bionic inspired surfaces for aerodynamic drag reduction on motor vehicle body panels. *Journal of Zhejiang University: Science A*.

[15] Okano T., Koishi M. Hydroplaning simulation using MSC. dytran.

[16] Liang C., Wang G., An D. F., Ma Y. W. (2013). Tread wear and footprint geometrical characters of truck bus radial tires. *Chinese Journal of Mechanical Engineering*.

[17] Isam J., Ali R., Vincent E. Tire tread pattern analysis for ultimate performance of hydroplaning.

[18] Zhao Y., Tan H. H., Zhang B. (2002). A high-resolution characteristics-based implicit dual time-stepping VOF method for free surface flow simulation on unstructured grids. *Journal of Computational Physics*.

[19] Ridluan A., Eiamsa-ard S., Promvonge P. (2007). Numerical simulation of 3D turbulent isothermal flow in a vortex combustor. *International Communications in Heat and Mass Transfer*.

